# Maximal Dependence Capturing as a Principle of Sensory Processing

**DOI:** 10.3389/fncom.2022.857653

**Published:** 2022-03-25

**Authors:** Rishabh Raj, Dar Dahlen, Kyle Duyck, C. Ron Yu

**Affiliations:** ^1^Stowers Institute for Medical Research, Kansas City, MO, United States; ^2^Department of Anatomy and Cell Biology, University of Kansas Medical Center, Kansas City, KS, United States

**Keywords:** object recognition (OR), computational modeling, invariant representation, sparse recovery (SR), redundancy reduction, redundancy capturing, sparse coding, grandmother cell

## Abstract

Sensory inputs conveying information about the environment are often noisy and incomplete, yet the brain can achieve remarkable consistency in recognizing objects. Presumably, transforming the varying input patterns into invariant object representations is pivotal for this cognitive robustness. In the classic hierarchical representation framework, early stages of sensory processing utilize independent components of environmental stimuli to ensure efficient information transmission. Representations in subsequent stages are based on increasingly complex receptive fields along a hierarchical network. This framework accurately captures the input structures; however, it is challenging to achieve invariance in representing different appearances of objects. Here we assess theoretical and experimental inconsistencies of the current framework. In its place, we propose that individual neurons encode objects by following the principle of maximal dependence capturing (MDC), which compels each neuron to capture the structural components that contain maximal information about specific objects. We implement the proposition in a computational framework incorporating dimension expansion and sparse coding, which achieves consistent representations of object identities under occlusion, corruption, or high noise conditions. The framework neither requires learning the corrupted forms nor comprises deep network layers. Moreover, it explains various receptive field properties of neurons. Thus, MDC provides a unifying principle for sensory processing.

## Introduction

The world is organized into objects that form the basis of our daily experience. Objects can be assigned meanings from their associations with others and can predict future events. Object recognition is a subject of intensive study in neuroscience, machine vision, and artificial intelligence. It refers to a collection of problems that involve identifying an object from varying input patterns. The most studied object recognition tasks include image segmentation, size and location invariance, representation of 3-D images, identifying occluded objects, and object classification.

Object recognition is a hard problem because a given object can turn up in numerous appearances, can be obscured and occluded, yet the brain readily recognizes it (Ullman, 1996; Riesenhuber and Poggio, 1999b; DiCarlo and Cox, 2007). The prevailing framework of object recognition divides the problem into two subproblems: representation and decision (DiCarlo and Cox, 2007). Objects are thought to be represented in the cortical regions, which are then distinguished and properly classified. With this division are two sets of difficulties. The first is to identify the computational rules that allow the transformation of sensory inputs into accurate and invariant representations. The canonical framework is vision-centric, based on our understanding of highly advanced visual systems such as those in primates. Under this framework, object representation is achieved through hierarchically organized networks of neurons (Riesenhuber and Poggio, 2000; Serre, 2014). In the meantime, it is also premised on efficient coding, where individual neurons transmit information efficiently. There are no intrinsic computation rules that allow the mapping of variant inputs onto the same representation. The second set of difficulties is related to resolving the ambiguities in the representations and determining whether a representation belongs to a specific object or a class of objects. The canonical solution to this problem is for a network to be trained using a large set of examples to achieve statistical robustness in object identification. How different representations are properly classified based on statistical learning has created difficult challenges (Chen et al., 2018; Fiser and Aslin, 2001; Turk-Browne et al., 2005; DiCarlo and Cox, 2007).

Animals appear to solve object recognition seamlessly. They exhibit the acute ability to discriminate similar sensory inputs and identify objects in complex natural environments to guide their behaviors, often in a fraction of a second. Species with limited brain complexity can perform robust recognition. Juxtaposing the ease of most animals’ seemingly effortless ability to recognize objects and the difficulty of current models to solve this problem, one must ask, *why is object recognition hard*? We suggest that the vision-centric approaches have largely ignored the ethological need for object recognition from the perspective of animals. They do not provide the explanatory power to other senses, nor to visual systems that are less sophisticated but equally powerful in performing object recognition. The models may have incorporated specific elements from the visual system that are not needed for object recognition in general and created unnecessary complexity and difficulties in the field. Auditory and olfactory objects are recognizable entities with direct ethological relevance, but their input patterns are less defined than visual input. The neural circuits that process auditory or olfactory information do not have deep structures, but the recognition of odor or audio objects is nonetheless robust. Visual recognition is also strong even in species with simpler and less organized visual systems, such as rodents, arachnids, and insects. A general theory of object recognition must accommodate these systems.

In this article, we assess the assumptions, the framing, and key concepts in the current framework and offer new perspectives of the problem. We introduce an information-theoretical definition of object recognition and hypothesize that maximal dependence capturing is a general principle in sensory processing. We present evidence from mathematical simulations that the new framework allows robust object representation. At the same time, it also provides the power to interpret the firmly established experimental observations that form the basis of current models.

## A Critique of the Current Framework of Object Recognition

**FIGURE 1:**
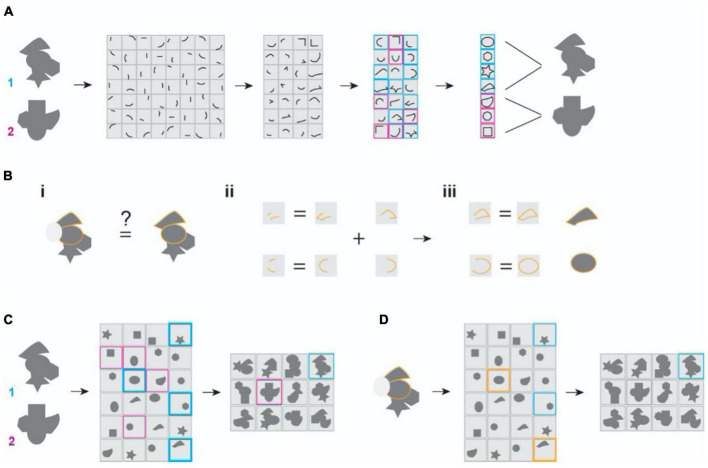
Illustration of the classic and the maximal dependence capturing frameworks for object representation. **(A)** Classic framework. Inputs are decomposed into independent features and are reassembled hierarchically into more complex combinations to represent separate objects. Two randomly shaped objects are depicted. Their representations at later stages are color-coded. Gray patches depict the receptive field properties of representation neurons. **(B)** The problem of missing parts. **(i)** Occlusion (depicted as the gray oval obscuring the object) needs to be associated with the un-occluded one to be identified. **(ii,iii)** The classic models require learning the occluded feature or feature combinations to be the same at every stage of processing. There is also a need to learn every corrupted form to reach robustness. **(C)** In the MDC framework, coding units capture structural relationships among the features and encode them as a whole. **(D)** In the MDC model, missing feature (grayed out area) does not affect the encoding because redundancy allows the same units to represent the features and the objects even when parts are missing. There is no need to learn from all corrupted forms.

The classic framing of the object recognition problem has been representational, i.e., neurons faithfully represent sensory features (Hubel and Wiesel, 1962, 1968). Object images are decomposed into elemental components that are processed across various stages (Figure 1A). This parts-based decomposition was the idea behind the computations performed in perceptron (Rosenblatt, 1957, 1958) and the theory of *recognition by components* (Biederman, 1985, 1987). Guided by brain anatomy and physiology, later theories propose the hierarchical assembly of elemental features into increasingly complex structures across multiple stages of sensory processing (Barlow, 1961; Atick, 1992; Riesenhuber and Poggio, 1999b; Ullman et al., 2002). In this set of theories, combinations of largely independent features form the basis of brain representations of the objects (Hubel and Wiesel, 1962; Marr, 1969; Marr and Nishihara, 1978; DiCarlo et al., 2012). The framework successfully explains the increase in size and complexity of receptive fields in the mammalian brains (Boussaoud et al., 1991; Rolls and Milward, 2000; Rolls, 2001). It can attain shift and scale invariance while representing 2-dimensional images (Fukushima and Miyaek, 1982; Anderson and Vanessen, 1987; Olshausen et al., 1993). Object representations can also be robust against occlusion (Fukushima, 2003, 2005; Johnson and Olshausen, 2005).

In more recent studies, numerous layers of convolutional and recurrent neural networks are trained to perform specific tasks (Yamins and DiCarlo, 2016a; Richards et al., 2019). These deep learning models can perform at levels that rival or exceed human performances (Cireşan D. C. et al., 2011; Cireşan D. et al., 2011; Sermanet and LeCun, 2011; Krizhevsky et al., 2012; Russakovsky et al., 2015). The success in deep learning and other AI approaches have reinforced the notion that the hierarchical architecture and computational rules associated with it may just be what neuroscientists have been looking for. Indeed, recent efforts have been comparing deep neural networks (DNNs) with brain structures in performing certain tasks (Lee et al., 2007; Yamins and DiCarlo, 2016b; Ponce et al., 2019; Bao et al., 2020).

However, building something to perform a similar function does not mean we have reproduced biology – an airplane uses completely different mechanics from birds to fly. Questions arise whether the DNNs recapitulate the inner working of the brain and if there is a need to engineer less artificial intelligence (Sinz et al., 2019). We believe that there are fundamental conceptual problems inherent in the current framework. Here, we wish to identify these problems. We do not clearly distinguish between the two fields because both the brain and AI models adopt the same assumptions and framing.

### One Problem, Disparate Solutions

To individual organisms, object recognition is for the purpose of determining the presence of an object. In computation models, the task has been divided into multiple problems for the brain to solve. The solution that addresses a specific problem often does not apply to others. For example, dividing the cognitive task into two distinct operations creates incompatible solutions. In the first operation, sensory features belonging to an object are hierarchically represented. Various features associated with the same object must be segregated from the background elements, and they need to be bound together (Von der Malsburg, 1995) (Riesenhuber and Poggio, 1999a; Singer, 1999). As such, the representation of objects also must solve the “binding” problem, which differs from the segmentation problem that assigns features to the proper objects when ambiguity arises (Treisman, 1999). Studies have suggested utilizing the temporal synchrony of neurons (Singer, 1999; Von der Malsburg, 1995) or non-linear maximum pooling of their activities to solve these segmentation and binding problems (Riesenhuber and Poggio, 1999a,b).

In the representation stages, perspective invariant representation is also to be achieved. The main model maps various perspectives of a 3D object to a stored standard view to achieve perspective invariance (Ullman and Basri, 1991; Ullman, 1996, 1998). However, at this stage, activities of view-tuned neurons are combined linearly as weighted summation (Poggio and Edelman, 1990; Poggio and Girosi, 1990) rather than non-linear maximum pooling proposed for the binding problem.

In the second operation, the process of discrimination and classification requires yet another set of rules. These rules are mostly associated with statistical learning, for example, manifold learning to disentangle representations of the same objects from others (Chen et al., 2018; DiCarlo and Cox, 2007). Moreover, the learning process requires labels for the classes. In the artificial networks, only the readouts at the final stages contain information to unambiguously classify or identify the objects (Krizhevsky et al., 2012; Goodfellow et al., 2016). This design does not have a parallel in the brain.

The framing of object recognition as a two-step process is problematic not simply because of the disparate solutions required. It has fundamental flaws in the assumptions. First, the various forms of input corresponding to the same object will have as many representations. There is no *a priori* label to tell that these patterns belong to the same object and allow the type of learning in current neural network models. Second, even if such a mechanism exists, it requires storing class information of individual objects separate from the representations themselves, which is a problem in and of itself. Finally, it is unknown where in the brain the separation between representation and classification takes place.

### The Conundrum of Hierarchical Assembly

Since Hubel and Wiesel first proposed hierarchical organization to explain the complexity of the receptive field properties observed in the visual pathway, the concept has become a cornerstone in understanding visual processing (Hubel and Wiesel, 1962; Marr, 1969; Marr and Nishihara, 1978; DiCarlo et al., 2012). Furthermore, studies of the visual processing streams have revealed shape-specific cells in high-order centers like V4 and IT. These cells are selectively tuned to faces or objects (Gross et al., 1969; Tanaka et al., 1991; Gross, 1992; Tanaka, 1992) and are involved in their recognition (Damasio et al., 1990; Damasio and Damasio, 1993). However, while physiological evidence is consistent with the hierarchical model, there is little anatomical evidence to demonstrate the progressive integration of elemental features along the hierarchy. Although cells in V1 have larger receptive fields than the retina, there is no obvious difference between V1 and V2 (Van den Bergh et al., 2010). In rodents, the receptive field is already large in V1, where the neurons’ spatial tuning can be as large as 34 degrees (Van den Bergh et al., 2010). Nor is there strong evidence indicating that the cortical neurons perform stepwise integration. In fact, Felleman and Van Essen have argued that “there is no *a priori* reason to restrict the notion of hierarchical processing to a strictly serial sequence” and “any scheme in which there are well-defined levels of processing can be considered hierarchical” (Felleman and Vanessen, 1991). Indeed, it appears that each stage is reorganizing the input patterns for specific purposes (Bruce et al., 1981; Desimone et al., 1984; Tsao and Livingstone, 2008).

Nevertheless, many hierarchical models of object recognition rely on serial integration to achieve object representation (Fukushima and Miyaek, 1982; Anderson and Vanessen, 1987; Olshausen et al., 1993). These models share a conundrum with regard to how specific a cell should be in its response to sensory features. Experimental observations indicate that high-order neurons can be highly specific (Quiroga et al., 2005; Ponce et al., 2019). Many cortical neurons respond robustly to specific stimulus patterns, but slight changes in input could greatly reduce their responses (Rolls, 1984; Tanaka et al., 1990; Tanaka, 1993, 1996). If neurons are highly selective, the number of neurons needed to accommodate the possible feature combinations is astronomical. The improbability of the Grandmother Cells best illustrates this issue (McCulloch et al., 1959; Konorski, 1967; Barlow, 1995; Gross, 2002; Bowers, 2009). The concept of a grandmother cell refers to a neuron sitting at the top of a hierarchy to represent specific objects uniquely, even though it was initially raised as a singular addressable memory unit (Gross, 2002). While it is possible to create this type of highly selective cells, the requirement of generating specific cells that lead to the buildup of the grandmother cells is improbable.

In an alternate scenario, neurons can be less selective to avoid combinatorial explosion. Rather than relying on the highly specific responses, a population of less specific neurons can collectively encode the objects (Young and Yamane, 1992; Pasupathy and Connor, 2002; Chang and Tsao, 2017). However, it will be difficult to resolve ambiguities and distinguish similar input patterns in this arrangement. Recently it has been proposed that neurons at higher levels of visual processing do not represent specific feature combinations but encode individual axes in a high-dimensional linear space where each location corresponds to an object (Chang and Tsao, 2017). While such coding is possible, it may not be very effective in dealing with external and internal noises in the system. Deciding on the dimensionality of the object space creates another challenge. Moreover, there does not appear to be a need to code the entire space when only a few points in the space are relevant. This problem currently does not have a solution in the hierarchy model.

### Problem With the Efficient Code

Most sensory neurons are ambiguous in representing the physical or chemical properties of stimuli. Photoreceptors and cochlear hair cells respond to a range of light and sound spectra, respectively (Russell and Sellick, 1978, 1983; Crawford and Fettiplace, 1980, 1981; Goldsmith, 1990; Rodieck and Rodieck, 1998). In the olfactory system, multiple odorants activate individual sensory neurons (Ressler et al., 1993; Vassar et al., 1993; Mombaerts et al., 1996; Treloar et al., 2002; Mombaerts, 2006; Fantana et al., 2008; Ma et al., 2012). In addition, the neurons are noisy, and their response changes as they adapt to stimulus intensity, duration, or context (Stockman et al., 2006; Rieke and Rudd, 2009; Rudd et al., 2009). Such response characteristics create confound in deciphering the precise stimulus. Early processing stages appear to mitigate this confound. One theoretical foundation of the early sensory transformations is efficient coding (Attneave, 1954; Barlow, 1961). Adapted from Information Theory, efficient coding has focused on minimizing redundancy in information transmission by encoding independent features present in natural stimuli (Laughlin, 1981; Olshausen and Field, 1996; Bell and Sejnowski, 1997; Lewicki, 2002; Smith and Lewicki, 2006). Individual neurons tuned to these features serve as independent encoders that efficiently relay information about the surroundings (Field, 1994; Olshausen and Field, 1996, 1997; Bell and Sejnowski, 1997). Models based on this theory successfully explain the receptive fields of neurons in the retina and the primary sensory cortices (Laughlin, 1981; Olshausen and Field, 1996; Bell and Sejnowski, 1997; Lewicki, 2002; Smith and Lewicki, 2006). Also, as the theory predicts, the response properties of neurons in the retina, the thalamus, and the primary visual and auditory cortices conform to the statistics of natural stimuli (Barlow et al., 1957; Hartline and Ratliff, 1972; Srinivasan et al., 1982; Atick and Redlich, 1990; Dong and Atick, 1995; Olshausen and Field, 1996, 1997; Bell and Sejnowski, 1997; Simoncelli and Olshausen, 2001; Geisler, 2008).

However, these results are not without controversy. Neuronal recordings have revealed overlapping receptive fields and synchronized activity among the retinal ganglion and V1 cells in many species (Meister and Berry, 1999; Nirenberg et al., 2001; Reich et al., 2001; Puchalla et al., 2005; Pillow et al., 2008; Ohiorhenuan et al., 2010). Recent rodent studies show large spatial tunings of V1 cells and a much less organized primary visual cortex (Martinez et al., 2005; Niell and Stryker, 2008). In the mouse olfactory cortex, any given odorant activates multiple neurons not specific to the odorant (Poo and Isaacson, 2009; Stettler and Axel, 2009). These observations do not conform to efficient coding. They suggest a high correlation among neurons and redundant information transmission. Indeed, the presence of redundancy in neuronal responses has led Barlow to suggest that redundancy is useful for encoding object identities, although he has not proposed how the information is used (Barlow, 2001).

The efficient coding hypothesis, in fact, poses serious problems for cognitive robustness. An object is not merely a collection of features. The relationship among its features defines it. Encoding independent features remove information about these relationships from the sensory input. Consequently, the system faces a challenge to recover and store this information. The task becomes exceedingly difficult when occlusion, internal or external noise, or inactivity of neurons causes ambiguity in the input signal. Any inference of the absent fraction of the signal or the input is impossible without the relational information. Mechanisms like pattern completion can help in certain situations (Rao and Ballard, 1999; Lee and Mumford, 2003). However, these mechanisms require storing the relational information, a problem that does not have a ready answer.

### Unsolved Problems With Statistical Learning

To achieve robustness, the current frameworks of object recognition rely heavily on statistical learning or post-representational inference (Figure 1B). For example, a 3-D object can be aligned and associated with its multiple 2D views (Biederman, 1987; Ullman and Basri, 1991; Ullman, 1996). Similarly, corrupted and occluded inputs can be linked with their non-corrupted forms along a manifold through learning (DiCarlo and Cox, 2007). As such, recent neural network-based models utilize extensive training using many examples to identify objects from incomplete images or novel perspectives. Presumably, such comprehensive training reveals features and their relationships that facilitate robust recognition (Figures 1Bii,iii).

However, the robustness provided by statistical learning is *retrospective*, meaning that only the learned examples, or those closely resembling them can be identified with high accuracy. On the other hand, the animal brain can recognize objects with *prospective robustness*, which we define as the accuracy in identifying novel objects and their different forms without additional learning. Animal brains learn a new object and recognize it without having to experience all of its variations in form. This ability to use few examples and perform prospective recognition is missing in models based on statistical learning.

## New Perspectives of Object Recognition

Given the caveats of the current framework, we wish to offer new perspectives of object recognition. Specifically, we intend to establish a framework that considers the animal’s ethological needs and affords both retrospective and prospective robustness.

Before discussing the details of the framework, a short note on “objects” is necessary. Physical “objects” that we see and recognize are explicit in visual inputs. However, other sensory modalities also signal “objects” that serve similar functions. For example, an acoustic object comprises a specific combination of sounds with characteristic frequencies and durations (Griffiths and Warren, 2004). A distinct blend of odorants constitutes an odor object (Keller et al., 2007; Barwich, 2014, 2018, 2019; Smith, 2015). These “objects” are amorphous, but they signal the presence of their emitters. Further, an animal can trace them through directionality or concentration gradients. We believe that any discussion on object recognition must include these objects and should not just concern the visual ones.

### The Ethological Perspective

To understand object recognition, it is imperative to consider its behavioral and evolutionary purpose rather than the accuracy in representing the physicochemical properties of the stimuli (Burge, 2010). An object is meaningful to an animal when it is informative of its associated consequences. Accordingly, recognizing the presence of an object through its *identity* is paramount to the behavioral consequences. Once the animal establishes the object’s *identity*, it can act appropriately to increase the chances of its survival. Thus, the core objective of recognition is to determine objects’ identities to trigger proper behavior.

How does one determine object identities? All object features do not convey the identity information equally. Some features or feature combinations are more useful than others in identifying objects. Sensory organs capture information redundantly, which can be useful in eliminating input ambiguity in object identities. Accurately representing every physiochemical property of the stimulus may not be necessary. From this perspective, an animal does not need to know every detail about the object to identify it correctly.

In his seminal study, Barlow has described the “on-off” retinal ganglion cells in the frogs as “the detectors of snap-worthy objects” (Barlow, 1953, 1961). He argued that a prey fly at a reachable distance from the frog would exactly fill the receptive field of these cells and generate the most vigorous response in them (Barlow, 1953). Thus, the frog visual system can detect flies without representing every detail and reliably set off the hunting sequence. Indeed, a later study further characterizing these cells’ properties revealed hallmarks of perception rather than simple sensation (Lettvin et al., 1959).

However, the current models have lost this initial insight. While the idea of representing object identities without the specifics is not entirely new (Marr and Nishihara, 1978; Barlow, 1989; Logothetis et al., 1994; Marr, 2010), the principal focus in current models is on accurate feature representation and reproducing the receptive field properties of the cells. Representation of object identities is pushed to the top of the hierarchal representation structure, and the hierarchy and deep structures have become obligatory for object recognition.

Arguably, object representations at different levels of hierarchy serve different purposes. If a part of the nervous system can accumulate sufficient information from the features or combination of features to guide behaviors, it has achieved object recognition. It does not need representation at the top of the hierarchy. Frogs can snag prey using just “on-off” cells in their retina. Moreover, there is no need for processing through a deep structure of neural networks. Relatively shallow structures process odor information in species across phyla. The insect and mammalian olfactory systems have two processing stages: the antennal lobe and the mushroom body in insects, and the olfactory bulb and the olfactory cortices in mammals. This shallow structure nonetheless allows robust recognition of odor objects to guide behaviors (Kobayakawa et al., 2007). Lastly, the early stages are not required for processing at the later stages. For example, the primary auditory cortex is not required for speech recognition (Hamilton et al., 2021). Thus, encoding object *identity* does not require an accurate representation of stimulus *features* along a hierarchy.

### An Information-Theoretical Perspective

What is sufficient information to determine object identity? Multiple objects often share the same features; therefore, these features cannot uniquely identify an object. The uniqueness of an object resides in the specific combination and the structural relationships among these features (Hoffman and Richards, 1984; Biederman, 1987; Hummel and Biederman, 1992). For example, recognizing a predator in various concealments or camouflages is possible because the relative configuration of the colors, spots, and shapes, though only visible partially due to the concealment, is adequate for its identification. In other words, the information sufficient for recognizing an object is embedded in feature combinations that considerably reduce the uncertainty about it.

Notably, for a given object, there can be multiple feature combinations that uniquely identify it. From the information-theoretical perspective, these feature sets provide redundant information about the object, which can be useful in resolving ambiguities. Therefore, any sensory processing framework must address encoding the most informative structural components and using redundancy to generate consistent object representations across different experiences.

To better understand the informativeness of features about objects, suppose we need to identify N objects, each defined by a unique combination of M features. For a given object *O_i_*, let its full set of features be *f*_*full*_. Assuming a condition where only a subset *f*_*subset*_ of *f*_*full*_ is available to the system, the following relation with regard to the entropy *H* holds:


(1) $$H\left(O_{i}|f_{subset}\right)=H\left(f_{full}|f_{subset}\right)=H\left(f_{full}\right)-I\left(f_{full};f_{subset}\right)$$

i.e., the uncertainty in predicting the full set of features or the object given an input subset decreases as the mutual information (I) between the given subset and the full set increases. This mutual information is the informativeness of the given subset of features about the object. From this information-theoretical perspective, *object recognition is achieved when the subset of features is maximally informative and uncertainty about the object vanishes*. At this point, the subset unambiguously informs the brain of the object’s presence. Multiple feature combinations can be equally informative about the object; therefore, multiple ways of representing the same object based on these combinations are possible.

With this definition, we propose that the problem of object recognition be stated as finding the computational rule that allows the neurons to robustly encode object identities using the most informative feature combination. We next develop this idea to propose the maximal dependence capturing principle and provide a mathematical solution to the problem.

### The Maximal Dependence Capturing Principle

We can express the informativeness of features as the information provided by the representation units (the encoders). If there are *K* encoder{*x*_1_ …, *x*_*j*_ …, *x*_*K*_}, each capturing a different substructure of the object *O*, then the mutual information between encoder *j* and the object *I*(*O*; *x*_*j*_) is the same as the informativeness of the sub-structure about the object. In noiseless conditions


(2) $$\sum_{j}I\left(O;x_{j}\right)\geq I\left(O;X\right)$$

where *X* is the representation of the object.

This relation shows that in a noiseless situation the sum of all information about the object from individual encoders will be more than the information about the object from its entire representation. This extra information can help resolve ambiguities. Further, fewer encoders can be sufficiently informative when the system maximizes the mutual information between the object and individual encoders. The higher the mutual information, the fewer the encoders are needed, and the more robust the encoders can be in identifying an object.

Most frameworks of sensory processing consider the encoders to be independent. This arrangement minimizes the mutual information between them, creating situations where individual encoders are not informative of any object. In contrast, we suggest that individual neurons capture maximum information about individual objects and are not independent. We refer to this as the maximal dependence capturing (MDC) principle because mutual information measures dependence. Further, we propose sensory processing to follow this principle.

This principle, as we show, leads to a sensory coding framework that can enable robust object recognition (Figures 1C,D). In this framework, neurons do not serve as independent encoders but encode the structural relationship among sensory features. It is not difficult to see that if a neuron captures the entire object structure, it can uniquely represent it. Other neurons may detect the object’s substructures and convey redundant information, but they are not needed. Using a mathematical model, we demonstrate that a specific form of sparse coding enables capturing such dependence and generates unique representations of the same object in various forms. This framework can achieve prospective robustness without the requirement of deep layers or statistical learning. We show that the framework is adaptive to object sets and can naturally lead to simple cell-like receptive field properties that have been characterized as independent encoders.

From this framing, several disparate problems can be treated as the same. Corruption and occlusion, for example, are problems of recovering full identities from partial signals. If neurons encode the dependence between the visible features and the object, missing parts can be inferred from the dependence to effectively solve the occlusion problem. As the dependence does not change with scale or location, the corresponding invariances can be achieved. The same holds even for 3-D object representations. Since each 2-D view can be considered a unique combination of a subset of an object’s features, multiple 2-D views can provide the same identification. Therefore, the view angle problem becomes whether a particular view of an object contains sufficient information to determine its identity. From the information-theoretical perspective, the solution to these disparate problems is the same: capturing the most information about the object as feature combinations. Moreover, as we show below, the activity of the most informative units can be maintained the same even when the input pattern is incomplete. This characteristic removes the inference requirement, thereby allowing the same set of rules to accommodate both representation and classification.

## The Model

We find that during the linear transformation of input patterns to representations, individual neurons can get tuned to comprehensive input structures if we constrain the representations to be sparse and make the transformation process non-negative. The sparsity constraint ensures that any object representation comprises a minimal number of active neurons and is maximally distinct from others. Non-negativity in transformation prevents encoding inputs as differences of structures. This type of coding eliminates the chances of neurons getting tuned to superpositions of multiple objects. Also, a non-negative representation is biologically more meaningful because action potentials are positive signals.

The specific objective function that we optimize to attain the desired transformation for a finite set of objects is


(3) $$\underset{\Phi ,A}{argmin}{||\text{X}-\Phi \text{A}||}_{2}^{2}+\lambda {||A||}_{1}subjectto\Phi \geq 0and\text{A}\geq 0$$

Here *X* is a matrix of inputs, and *A* is a matrix of corresponding representations. Φ is a matrix with columns corresponding to the tuning properties of the representational neurons. It serves as the basis set for representations and comprises the inputs’ informative structures. We refer to it as the *dictionary* according to convention.

The first term in the objective function measures the difference between the input structure and the structures captured in their representations. Minimizing it ensures that the representations reflect most of the input structures. The second term is a measure of representation sparseness. It serves as a penalty on the total activity of neurons in a representation. Note that the intention is to reduce the number of active units in a representation, i.e., the *l*_0_ norm, to minimize representation overlap. Mathematically, however, an analytical solution to *l*_0_ minimization is not possible. Therefore, we minimize neurons’ total activity in representations (the *l*_1_ norm of representations). Also, note that we set the representation dimension to be larger than the inputs to achieve sparseness. In this setting, the transformation conforms to the observation that higher brain centers often possess several folds more neurons than the sensory organs.

The optimization function is similar to those employed to capture the natural scenes’ independent components (Olshausen and Field, 1996, 1997; Simoncelli and Olshausen, 2001; Geisler, 2008). However, we use this objective function in a different context. Instead of identifying the independent features based on the statistics of the entire input space, the goal here is to represent a limited set of inputs by capturing their most informative structures. Furthermore, the non-negativity and the sparsity constraints force individual neurons to tune to comprehensive input structures. Without non-negativity, the tuning properties of neurons can be arbitrarily complex (Olshausen, 2013).

### Dependence Capturing

**FIGURE 2:**
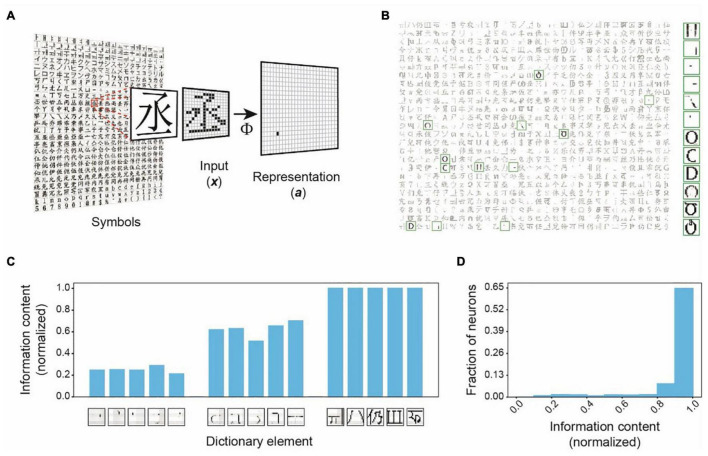
Dependence Capturing by the MDC framework. **(A)** Illustration of encoding in the MDC framework. Symbols from world languages are converted to 256 (16 × 16) pixel images (*x*) that are transformed into the activity of a set of 800 representation units (*a*) to encode symbol identities. An example of the process is shown using a character encoded by a single representation unit to indicate the ability to encode complex structural features. **(B)** Structures of the dictionary elements (receptive fields) learned from 1,000 symbols. Highlighted elements displayed in the larger size are most active while representing the inputs shown in Figure 3. Note the similarity among some of the elements. **(C)** Information contents of sample dictionary elements normalized to the maximum observed information content. While very simple structures are least informative about any object, more comprehensive structures are highly informative. **(D)** Distribution of the normalized information contents of the dictionary elements. Most of the structures are highly informative about specific objects indicating that the mathematical framework captures features that share maximum dependence with the inputs.

**FIGURE 3:**
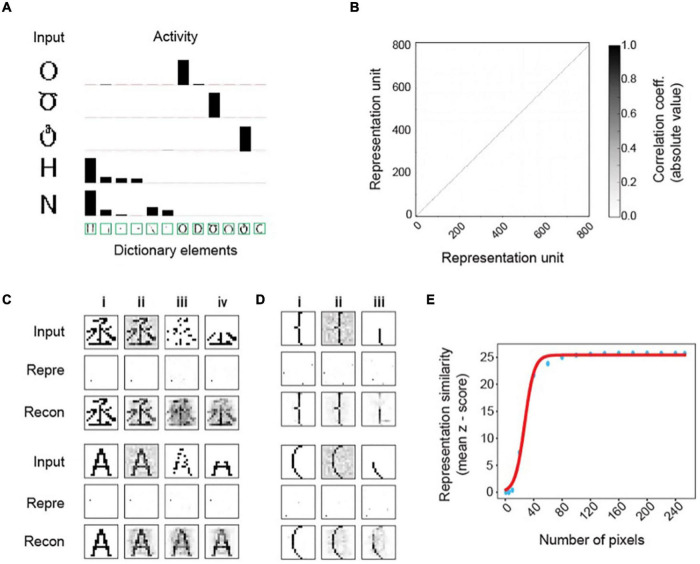
Invariant object representations in the MDC framework. **(A)** Representation of inputs by the activity of the dictionary elements. Only the most active ones are shown. The height of the bars indicates activity evoked by the input patterns. Note that the inputs only activate the dictionary elements that are most similar to them despite the similarity among the elements. Such coding results in very sparse input representations. **(B)** Correlation among representation units is minimal, and the pairwise correlation matrix of the representation units is an identity. **(C)** Representations of original **(i)** and corrupted inputs under noisy **(ii)**, pixel loss **(iii)**, and occlusion **(iv)** conditions. Representations of the corrupted signals (Repre) are similar to those of the originals. Reconstructed images (Recon) from the output units resemble the original symbols. **(D)** Example of two highly similar symbols being distinctly and robustly represented. The original input signals **(i)** corrupted by noise **(ii)** or occlusion **(iii)** are transformed to output activities that are similar to each other. Reconstructed images recover the original signals. **(E)** Z-scored similarity (specificity) between representations of corrupted and original signals as a function of total pixels in the input layer [randomly selected as in panel **(Ciii)**]. Scores calculated with different pixel numbers (blue dots) is fit to a sigmoidal curve (red line).

We illustrate this framework’s key characteristics by encoding binary symbol images from world languages (Figure 2 and Supplementary Figure 1A). With 256 input and 800 output units, the representations in these simulations are dimensionally expanded (Figure 2A). Learning to represent 1,000 symbols results in dictionary elements containing local and global structures (Figure 2B). Structures of individual dictionary elements contain varying information about different inputs. The localized structures are less informative about any input, but the comprehensive structures are unique to specific inputs and contain the most information about them (Figure 2C). We plotted the histogram of the dictionary elements’ maximum information contents for any input (Figure 2D). The histogram is heavily skewed toward larger values, indicating that the framework successfully captures the highly informative structures. An interesting observation in these simulations is that multiple dictionary elements are structurally similar. For example, several dictionary elements shown in Figure 2B have the same oval-like shapes. These dictionary elements are utilized in distinctively representing very similar input symbols (Figure 3A). These symbols have subtle differences, which are captured in these matching dictionary elements. Thus, the finding demonstrates that the MDC framework does not just capture the distinguishing structures. It allows the redundant encoding of features shared by multiple objects. The computation in the framework can successfully extract complex structures naturally present in the stimuli without creating arbitrary or overly complicated dictionary elements.

### Prospective Robustness in Invariant Representation

We next test whether the MDC framework can encode objects distinctively, especially in conditions of noise and corruption. In classic frameworks, neural network models require deep layers to enhance robustness. The MDC framework captures comprehensive input structures, and we expect it to be robust against corruption without the deep layers. To obtain the representations of corrupted input patterns using the learned dictionary, we optimized the following objective function under the non-negativity constraint:


(4) $$\text{minimize}{||a||}_{1}\mathrm{subject}\mathrm{to}{||\text{x}-\Phi \text{a}||}_{2}\leq \varepsilon$$

The optimization ensures that the MDC framework utilizes the same computational rule for learning to obtain representation. Thus, it distinguishes itself from the hierarchical assembly, where the learning rule is separate from the transformation. This approach also contrasts with the previous approaches that use direct convolution of input with the dictionary to generate representations (Olshausen and Field, 1997; Rao and Ballard, 1999; Rozell et al., 2008; Lörincz et al., 2012).

The two-layer model based on our framework readily distinguishes highly similar patterns and represents them differently (Figure 3A). Representation neurons have minimum correlation (Figure 3B), and the symbol representations are sparse (Figure 3C). Whereas the correlation matrix of representation units is very close to identity, there are similar non-zero correlations among pixels in both the input and the dictionary (Supplementary Figures 1B,C). Resembling correlations indicate that the dictionary captures complete input structures.

Moreover, this framework achieves the desired invariance in representation. Without learning from corrupt examples, the model can transform inputs corrupted by Gaussian noise (Figure 3Cii), randomly missing pixels (Figure 3Ciii), or partial occlusion (Figure 3Civ) to representations identical to those of the uncorrupted input forms (Figure 3Ci). Reconstructing images by linearly combining dictionary elements restores whole symbols rather than parts (Figure 3C). Using the Z-score of the pairwise cosine distances between the representation of corrupted inputs and all learned symbols, we observe high specificity for the correct input-symbol pairs, indicating that the framework generates precise representations. Notably, the representations are sensitive to small differences in the input patterns. For example, two highly similar input patterns are represented differently and robustly under various corruptive conditions (Figure 3D). Monte Carlo simulations with randomly missing input units yield highly specific representations with as few as 60 (23.4% of the 256) units (Figure 3E). Thus, the computational framework fulfills the requirements of stability and sensitivity set forth by Marr and Nishihara (1978) without requiring deep layers or learning from many variable examples. Importantly, this example shows prospective robustness, as the stable representation of symbol identity does not depend on statistical learning through many corrupt examples.

### A Robust Code for Face Recognition

**FIGURE 4:**
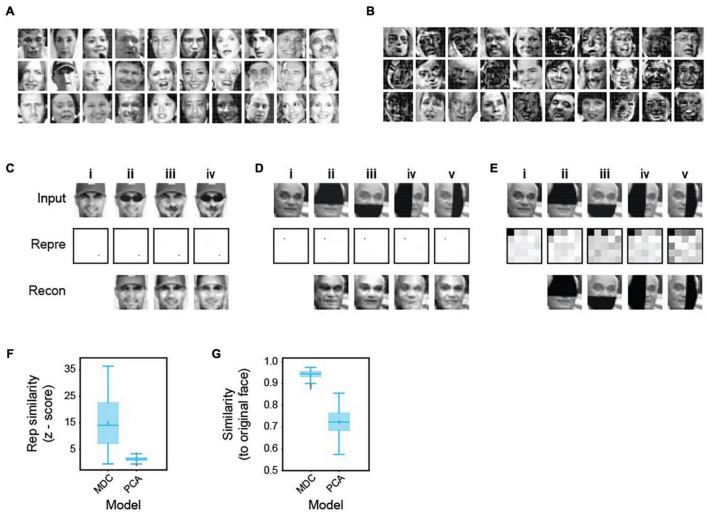
Robust representation of human faces. **(A)** Examples of face images in a library of 2,000 (1,000 male and 1,000 female) used to train a two-layer model. **(B)** Examples of dictionary elements learned from the face library. Note that they incorporate complex combinations of facial features but are not necessarily part or whole of any specific face. **(C)** Representation and recovery of faces with different alterations. A face **(i)** was altered to wear a pair of sunglasses **(ii)**, a mustache **(iii)**, or both **(iv)**. The altered faces’ representations were nearly identical to the original, even though these examples were not in the training set. Images reconstructed from the representations based on the dictionary were similar to the original images. **(D)** Representation and recovery of occluded faces. Four different occlusions of a face—top **(ii)**, bottom **(iii)**, left **(iv)**, and right **(v)** were generated. Representations of the occluded faces were highly similar to the original one **(i)**. Reconstructions also matched the original face. **(E)** Face identity was not preserved in representations based on PCA performed on the 2,000 training faces. Representations of original **(i)** and occluded faces **(ii–v)** were obtained in the principal space (the first 25 components are shown to highlight the differences). Representations of occluded faces are different from the original. Reconstructed images match the corrupted rather than original faces. **(F,G)** Specificity (Z-score similarity) of face representations **(F)** and cosine similarity between reconstructed and original images **(G)** were calculated for MDC and PCA models. 50 faces were chosen randomly from the training set to create the four occluded versions.

We next tested the two-layers model in encoding complex, non-binary signals such as human faces (Figure 4). We trained the model on 2,000 human faces (Figure 4A). The learned dictionary elements are composed of a complex assemblage of facial features, again suggesting that the algorithm captures complex structures present in the training set (Figure 4B). The face representations are stable, unique, and robust against common alterations such as headwear, facial hair, or eyewear (Figure 4C). The model produces nearly identical representations while transforming the same face with a mustache, a pair of sunglasses, or both. It maintains representation consistency even when we block half the input face in different positions (Figure 4D). Inversely reconstructed images from the representations are similar to the unadulterated faces (Figures 4C,D). Notably, the model achieves robustness from learning only 2,000 examples and without using any corrupted images. Achieving such consistency is in direct contrast to many approaches using variegated examples as training sets.

Moreover, the dictionary learned from the training set can be applied to an entirely new set of faces. We used it to obtain representations of facial images in the Yale face base, which contains 15 individual faces, 11 different lighting conditions, and facial expressions. The representation correctly categorized the faces according to the individuals (Supplementary Figure 2).

We compared our code against a recently proposed principal components-based face code (Chang and Tsao, 2017). Using dictionaries obtained through principal component analysis (PCA), the same face with different parts occluded generated different representations. Image recovery produces occluded but not uncorrupted images (Figure 4E). In contrast, images recovered from the MDC representations of corrupted inputs are similar to the original ones (Figure 4C,D). Quantification of specificity using Z-scores shows that our model generates highly specific representations (Figure 4F). PCA-based decoding does not exhibit such selectivity or similarity (Figures 4F,G). Thus, a face code based on the MDC framework is robust against corruption, whereas the one proposed before is not (Chang and Tsao, 2017; Stevens, 2018).

### Adaptive Nature of the Maximal Dependence Capturing Code

**FIGURE 5:**
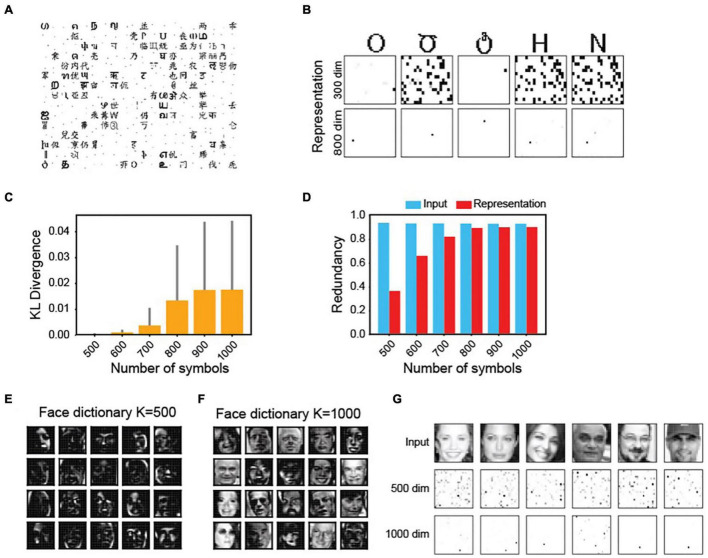
Complexity of tuning properties is determined by the number of objects and dimension of the representation. **(A)** The structures of dictionary elements for the symbols in Figure 2 with a 300-unit output layer. Compared with that of the 800-unit shown in Figure 2B, there are more localized features. **(B)** Representation sparsity increases with increased dimensions. **(C)** K-L divergence between pixel distributions in the input signal and in the dictionary element as a function of the number of symbols to be encoded. **(D)** Coding redundancies in input and output (representation) while encoding increasing numbers of symbols. **(E,F)** dictionary elements of faces with a 500-dimension **(E)** or 1,000-dimension **(F)** encoder-set. **(G)** Response elicited by faces are sparser with increased dimensions.

In the MDC framework, an increase in the number of objects can influence the dictionary elements’ structures. We explored the relationship by varying the numbers of encoded inputs (*N*) and representation neurons (*K*). With *N* fixed, at low *K*, dictionary elements are more localized (Figure 5A). The representations are less sparse and more active neurons encode the same symbol (Figure 5B). Increasing the number of neurons makes the response sparser.

Conversely, with a fixed *K*, an increase in the number of inputs causes a divergence of the dictionary element structures from the structure of inputs (measured using the K-L divergence (Kullback and Leibler, 1951) of pixel distribution between the input and the dictionary; Figure 5C). As more representation units encode each symbol, redundancy among them also increases (Figure 5D). At high *N*, the redundancy approaches that of the input, suggesting a decreased efficiency. The same observation holds for complex signals. The dictionary elements for faces are more localized at low *K* values, resembling local facial features (Figure 5E). At high dimensions, they become more complex and face-like (Figure 5F). More units are required to encode each face at lower dimensions (Figure 5G).

The MDC framework’s premise is that individual encoders maximally capture structures from the input signals, which results in complex dictionary elements. This characteristic appears to be contradictory to the localized receptive fields observed in primary sensory cortices. While the efficient coding hypothesis explains simple tuning structures as the independent components of natural inputs, we tested whether the MDC framework could also produce these localized receptive fields.

**FIGURE 6:**
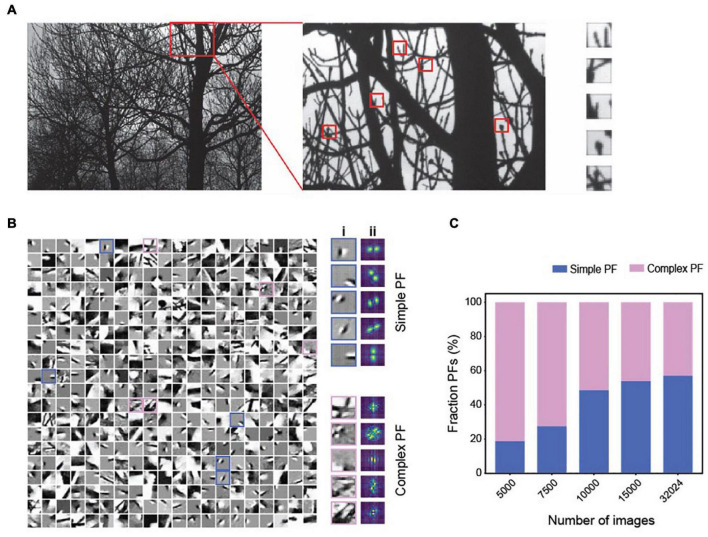
The emergence of simple and complex receptive fields from training with natural images in MDC. **(A)** Illustration of image patches derived from the natural scene. **(B)** Examples of structures of individual dictionary elements. Both simple (cyan) and complex (magenta) elements are observed **(i)** and can be determined using Fourier transforms of the structures **(ii)**. **(C)** As the number of training images increases, encoders’ tuning properties become more localized, and the percentage of simple PFs increases.

In our simulations, the tuning properties of neurons shift from being comprehensive to localized as the network encodes more objects. This trend suggests that the MDC framework captures simpler features when forced to encode more objects. In the visual system, the primary visual cortex relays all visual information. So, it must accommodate all visual objects. We reason that the overwhelming number of visual objects can force the cortical cells to adapt to the natural statistics of the visual input and tune them to the localized features. To test our reasoning, we trained the network with varying numbers of image patches taken from natural images (Van Hateren and van der Schaaf, 1998) (Figure 6A). Our method is like the approach of Olshausen and Field (Olshausen and Field, 1996; Olshausen and Field, 1997) but with non-negative constraints and a more limited number of training inputs. We parsed the images into positive and negative signals to simulate the On and Off channels in the mammalian visual system.

Training with up to 30,000 patches develops local, simple cell-like, and complex dictionary elements (Figure 6B). With a low number of training images, the dictionary elements are relatively complex (Figure 6C). Despite high correlations among the images, we observe minimal correlation among representation units (Supplementary Figures 3A,B). Increasing the training set size produces more dictionary elements with localized and orientation-selective projective fields similar to the receptive fields of simple cells in the mammalian primary visual cortex (Hubel and Wiesel, 1962) (Figures 6B,C). The simple cell-like dictionary elements resemble Gabor filters, as found in earlier studies (Field, 1987; Jones and Palmer, 1987).

Interestingly, complex receptive fields persist in all training conditions. The percentage of simple projective fields in the dictionary increases with the size of the training set. With a fixed set of training images, an increase in the encoding dimension reduces correlation among the encoding units but increases the correlations among dictionary elements (Supplementary Figure 3C). Thus, simple receptive fields conforming to the classic interpretation emerge when the model encodes many natural images under the MDC framework (Laughlin, 1981; Atick and Redlich, 1990; Dan et al., 1996; Lewicki, 2002; Smith and Lewicki, 2006). Importantly, complex tuning is always present in our model without any synthesis from the simple cells, as the classic model predicts (Hubel and Wiesel, 1962).

## Discussion

We propose that maximal dependence capturing is a general principle of sensory processing and an alternative to the redundancy reduction principle. The primary motivation behind redundancy reduction is to arrive at a factorial code for object representation to optimize information transmission (Barlow, 1961, 1989). However, a factorial code based on independent features is unsuitable for invariant coding, especially in corruption or occlusion cases. Hierarchical models learn complex feature combinations to achieve robustness and no longer use independent components for representations. With this “reduce and capture” strategy, where the model reduces redundancy among features before pooling them together by deep networks, the classic framework is self-conflicting and creates unnecessary problems. The MDC framework resolves the conflict with a “capture and reduce” strategy for redundancy. By assuming that dependence capturing is the essence of the sensory system, the coding units extract the most informative combination of features that uniquely identify specific objects. Though it makes representation redundant, sparse coding ensures that minimum correlation exists among the coding units. Thus, the framework obviates the need for a hierarchical assembly to associate features. It embeds individual features in complex dictionary element structures. Moreover, since the dictionary captures the dependence *a priori*, the framework does not require multiple corrupted forms to learn the associations.

The MDC framework’s unique characteristic is that the receptive fields capture highly informative structures about individual objects. With this characteristic, tuning of individual units can be very similar and correspond to multiple objects. As a result, correlations may develop in their responses. This characteristic appears to be antithetical to the notion of redundancy reduction, which demands individual encoding units to be as independent as possible. However, representations achieved under the MDC framework also satisfy a sparsity constraint. This constraint shrinks the activities of competing neurons and decorrelates individual units’ responses even when their receptive fields have high levels of overlap. Thus, the MDC framework allows the efficient encoding, but the coding is for object identities. It is done by capturing complex features.

The MDC framework makes specific predictions that can be tested through anatomical and physiological experiments. For example, it predicts that the connectivity required to achieve the orientation specific tuning of early neurons should be less organized than previous models predict (Hubel and Wiesel, 1962, 1968; Fukushima and Miyaek, 1982; Anderson and Vanessen, 1987; Olshausen et al., 1993; Rolls and Milward, 2000). Decorrelation among neurons through lateral connections is an essential feature of the MDC framework. Shutting down inhibitory lateral connections is expected to reveal highly overlapped receptive fields among the neurons. On the contrary, models based on connectivity alone would predict a much smaller degree of receptive field expansion. Moreover, while inhibition-mediated decorrelation is a common feature in the nervous system, our model would predict that cells with similar tuning properties are likely to have stronger mutual inhibitory connectivity. Manipulating the lateral connections would give insight about this prediction.

We have shown that the robust representation of object identity does not require deep network structures. Thus, this framework can explain robust object recognition in animals with less complex brain or sensory systems that do not possess complex hierarchical organizations. Nevertheless, hierarchical organization can arise to deal with increasingly complex stimuli during evolution. We suggest that as the animal needs to identify more objects, early processing can shift to encode localized features resembling the independent components. As we show, there is a relationship between the number of encoded objects and the complexity of the tuning properties, which allows both simple and complex receptive fields to develop under the same rule. When each neuron encodes the local association of features, multiple neurons are required to encode individual objects. The MDC framework allows subsequent levels to capture the global dependence among the local feature combinations. From the evolutionary perspective, the sensory systems have evolved to detect ethologically relevant signals. Analyzing environmental stimuli and parsing them into components of minimal redundancy is not necessary for this goal.

In a way, the MDC framework produces sparse distributed representations, which can account for some experimental observations, including the appearance of “grandmother cells” (Quiroga et al., 2005; Bowers, 2009; Rey et al., 2020). However, the previous models have mainly focused on reproducing the key features of neuronal tuning (Olshausen and Field, 1996, 1997; Bell and Sejnowski, 1997) and memory storage (Laurent, 1999; Palm, 2013). They do not provide a strong explanation for the change in the complexity of tuning properties along the processing stream. The MDC framework neither requires hierarchical assembly nor an account of all possible feature combinations. Since the encoders only capture the naturally occurring structure, the generation of “grandmother cell” like representation is a feature of the MDC framework. The cells, however, are not the traditional sense of “grandmother cells” because they do not sit at the top of any hierarchy.

A point worth noting is that the receptive fields of individual coding units resemble the objects themselves when the representations are sparsest. One may consider the coding scheme using these cells as a form of template matching (Burr, 1981; Buhmann et al., 1990; Yuille, 1991; Brunelli and Poggio, 1993). As we have shown, however, the MDC framework is not template-matching. The receptive fields may resemble whole structures of the objects, but they are not identical. Moreover, these receptive fields change as the coding units adapt to different numbers of inputs.

In this study, we have shown invariant representation for corrupted or occluded input patterns. Representation invariance takes many forms. Invariant representations result from scale, translational, and affine transformations are common. Although we have not explored these forms, we believe the framework will allow easy incorporation of these transformations without evoking additional mechanisms. As the name of the MDC principle indicates, dependencies among the features are captured by neurons. The dependencies, and the informativeness of features, do not change with linear transformation. Thus, the framework will naturally generate covariant receptive field properties that are thought to enable invariant representation of the visual stimuli at higher levels of processing (Lindeberg, 2013, 2021).

Likewise, understanding temporal dependencies between objects and events and the ability to predict future is fundamental to survival of the organisms. More recent studies have focused on efficient representation of moving images (Rao and Ballard, 1997; Rao, 1999; Srivastava et al., 2015), whereas some have produced models encoding features that best predict future (Bialek et al., 2001; Palmer et al., 2015; Salisbury and Palmer, 2016). Although we have not explored the effectiveness of the MDC framework in capturing temporal redundancies, it is in principle achievable. At the minimum, the framework can be combined with other models to incorporate temporal dependence capturing. For example, Singer et al. introduces a two layered feedforward network to predict future frames of movies (Singer et al., 2018). It should be straightforward to achieve invariant representations of static frames, which can be utilized together with the Singer model to predict future frames more accurately. Alternatively, a unified model, which may involve multiple layers, can capture not only spatial but also temporal dependencies to predict environmental stimuli in the spatiotemporal domain.

In summary, this work offers a novel perspective of object representation. We propose that the sensory system should utilize the most informative structures from objects as the basis of their representations. The maximal dependence capturing principle allows neurons to capture these structures by learning the relationships among features that identify the objects. This type of learning eliminates the need for an analytical step to break down objects into its composing features and the need of the classic hierarchical assembly where brain representations of objects are built sequentially from their elementary features. Learning is possible without the deep structures or large training set. These characteristics of our framework make it generalizable to any system irrespective of its complexity. It achieves the main objective of object recognition that is to establish the identities of objects and use the information to predict future events. Taken together, maximal dependence capturing offers a single framework to achieve robust object representation and explain seemingly contradictory observations on the neurons’ receptive field properties in different brain hierarchies.

## Materials and Methods

### Learning Algorithm

Dictionary learning is treated as a blind source separation (BSS) problem (Comon, 1994; Comon and Jutten, 2010). An input signal is modeled as the response of *M* primary encoders. In the case of images, *M* = *m*_1_⋅*m*_2_, where *m*_1_ and *m*_2_ are the horizontal and vertical dimensions of the images. A set of N signals is presented for training as an input matrix *X* ∈ℝ^*M*×*N*^, representing the response of *M* pixel to *N* patterns. The matrix *X* is then factorized into two matrices *A* and Φ, so that *X* = Φ*A*. Here, *A* ∈ℝ^*K*×*N*^ is the matrix representation of *N* patterns in a *K* dimensional basis set defined by Φ ∈*R*^*M*×*K*^.

To get the factor matrices through BSS, we imposed restriction on *A* to be sparse. The measure of sparsity was chosen to be *l*_0_ norm, but the solution is achieved through minimizing *l*_1_ norm. In addition to this sparsity constrain, we demanded both *A* and Φ to be non-negative.

Several possible BSS algorithms could result in an appropriate matrix decomposition under the given constraints (Hoyer, 2002, 2004; Rapin et al., 2013a; Allen et al., 2014). In particular, we used non-negative blind source separation algorithm nGMCA (Donoho and Elad, 2003; Rapin et al., 2013a,b). When a *l*_1_ measure of sparseness is used, then the sum of the absolute values of coefficients of *A* is minimized. The minimization problem takes the form of:


$$\underset{\Phi ,A}{argmin}\frac{1}{2}{||\text{X}-\Phi \text{A}||}_{2}^{2}+\lambda {||A||}_{1},subjectto\text{A}\geq 0;\Phi \geq 0$$

Thus, the process to solve this problem requires the minimization of the *Frobinius* norm difference (i.e., the Euclidean Distance) between the two sides of the equation and the minimization of the *l*_1_ norm.

Each time BSS is performed, the Φ matrix was seeded with random numbers. Optimization was performed until convergence or when predefined number of iterations was reached.

### Sparse Coding

Once the dictionary Φ is learned, any input pattern can be transformed into its corresponding representation. Transformation of input patterns is the process of finding the representation *a* that satisfies the equation: **x** = **Φa**. In our case, the dimension of the representational layer is chosen to be higher than that of the input layer, i.e., *K > M*. Here, decoding becomes an under-determined problem. Theories developed independently by Donoho (Donoho and Elad, 2003; Donoho, 2006a,b), and by Candes and Tao (Candes and Romberg, 2005; Candes et al., 2006; Candes and Tao, 2006) show that a unique solution can be obtained by imposing a sparseness constraint to the equation when solving the optimization problem. The most common use of sparsity definition includes *l*_0_ and *l*_1_. In our approach we perform *l_1_* minimization to solve:


$$\min{\left\|a\right\|}_{1}\;\mathrm{subject to}\;{\left\|x-\Phi a\right\|}_{2}\leq \varepsilon$$

The *l*_1_-minimization problem can be implemented by a standard convex optimization procedure, which can be found in several publications (Chen et al., 2001; Boyd and Vandenberghe, 2004; Candes and Tao, 2005; Donoho, 2006b; Donoho et al., 2012).

### Redundancy Measurement

To measure redundancy in encoding objects, we treated the objects as following a uniform distribution, i.e., ℙ(*Q_i_*) = 1/*N*, where *N* is the total number of objects. The entropy of the ensemble of the objects is therefore *H*(*O*) = *logN*. We then calculated the capacity of the input unit set (*C*) using the probabilities of occurrence of each encoder, ℙ(*x*_*i*_ = 1) = *p*_*i*_:


$$C=\sum_{i=1}^{M}p_{i}log\frac{1}{p_{i}}+\left(1-p_{i}\right)log\frac{1}{\left(1-p_{i}\right)}$$

Redundancy was calculated as


$$R=1-\frac{H\left(O\right)}{C}=1-\frac{logN}{C}$$

The redundancy for representational units was calculated in a similar way, the only difference being that the representations were converted to binary forms using a Heaviside step function so that their *l*_0_ norms could be considered while calculating probability of occurrence of individual encoders.

### Kullback–Leibler Divergence Between Dictionary and Images

We used the Kullback–Leibler divergence (KL Divergence) to quantify the structural differences between symbols and dictionary elements. KL divergence [*D*_*KL*_(ℙ||ℚ)] is a measure of information gained when a posterior probability distribution ℙ is used to calculate the entropy instead of the prior distribution ℚ. Denoting ℚ to be distribution over the states of a single pixel in symbol space and ℙ to be the distribution over states of the same pixel in dictionary space, *D*_*KL*_(ℙ||ℚ) measures the information gained in considering the pixel to be coming from a dictionary element rather than symbols. A low divergence for all the pixels indicates that there is no gain in information if we consider any pixel to be coming from dictionary, indicating that the structure of the dictionary elements is same as structure of the symbols.

To calculate the distribution over the states of pixels in the dictionary space, all dictionary elements were binarized using a Heaviside step function. Probability of occurrence of individual pixels was calculated based on the number of dictionary elements in which the pixel is active. For instance, if a particular pixel *x_i_* was active in *n* out of *K* dictionary elements, then the probability of occurrence of pixel *x_i_* was calculated as


$$\mathbb{P}\left(x_{i}=1\right)=\frac{n}{\text{K}}$$

Probability of occurrence of the same pixel in symbol space is calculated based on the number of symbols *m* in which it is active i.e.,


$$\mathbb{Q}\left(x_{i}=1\right)=\frac{m}{\text{N}}$$

Here *N* is the number of symbols being encoded. Finally, the KL Divergence between the two distributions is calculated as


$$\begin{array}{c} D_{KL}\left(x_{i}\right)=\mathbb{P}\left(x_{i}=1\right)log\frac{\mathbb{P}\left(x_{i}=1\right)}{\mathbb{Q}\left(x_{i}=1\right)}\\ \;+\left(1-\mathbb{P}\left(x_{i}=1\right)\right)log\frac{\left(1-\mathbb{P}\left(x_{i}=1\right)\right)}{\left(1-\mathbb{Q}\left(x_{i}=1\right)\right)} \end{array}$$

### Specificity Calculations

To quantify the specificity of a representational vector in representing the original object, we computed Z-scored similarity. Cosine similarity score between the representation of the test object (*a*_*test*_) and all objects in the training set (*A*_*training*_) were calculated and Z-scored. A high Z-score indicated high similarity between the representations of the test object and a particular object in *A*_*training*_. In the figures, we plot the Z-scores for altered images with their unadulterated counterpart, which show high specificity in representing the original object.

### Simulating Corrupted Signals

To test the robustness of object representation by the MDC framework, signals from the training set were selected and corrupted. The corrupted signals were subject to sparse decoding to generate their representations, which were then compared with those of the signals in the training set. We performed the following three types of corruption:

Noise-added corruption: we introduce noise by adding a Gaussian i.i.d. matrix 𝒩 of varying standard deviation to the input matrix *X*. i.e., *X*_𝒩_ = *X* + 𝒩, where *X*_𝒩_∈ℝ^*M*×*N*^, is a matrix representation of noisy input. For Monte Carlo analysis, as described below, each time a simulation was performed, a different noise matrix 𝒩 was introduced.

Pixel corruption: For a given signals, a fraction of the *M* pixels was selected from the input. Their values were maintained whereas the coefficients of the rest were set to zero.

Occlusion: For images, a contiguous set of pixels were selected, and their values were set to zero.

### Monte Carlo Analysis

We performed Monte Carlo simulations by applying pixel corruption to the input signals and varying the number of corrupted pixels. 100 random sets (numbers varied from 2 to *M*) of pixels were selected. Using each of these randomly chosen sets, we performed sparse decoding to generate representation of the input patterns.

### Input Identification

To calculate the correct identification of the object, we used the representation of each input in the training set as a library. The representation of each corrupted signal was compared with that in the library and cosine distances were computed. An input pattern was considered correctly identified if the cosine distances between its representation and that of the original signal was minimum (smaller than with representation of other patterns).

### Projective Fields Generation From Natural Images

The mammalian visual systems process visual information in On and Off channels. On channel images were the normal images whereas Off channel images were the inverted images. To simulate parallel processing of the two channels, the On and Off images were concatenated along the rows and dictionary elements were generated by performing BSS on the concatenated matrix. The projective fields were constructed by superposing the On-channel portion of the dictionary element with the negative of Off-channel portion of the dictionary element.

### Data

Symbols: A set of 1,000 symbols from word languages were obtained and digitized to 16 × 16 pixel arrays.

Natural images: Natural scenes from Van Hateren data base (Van Hateren and van der Schaaf, 1998) were digitized as grayscale pictures. Image patches of 16 × 16 size were randomly selected from the images. A total of 3,000 patches were used to form a training set.

Facial images: For face recognition, 2,000 frontal faces were obtained from Google search of publicly available images, trimmed and resized to 25 × 25 pixels. The Yale Face Database is obtained from http://cvc.cs.yale.edu/cvc/projects/yalefaces/yalefaces.html.

## Data Availability Statement

The raw data supporting the conclusions of this article will be made available by the authors, without undue reservation.

## Author Contributions

CRY conceived the idea and supervised the research. CRY and RR developed key concepts and co-wrote the manuscript. RR and DD performed analyses and modeling. KD generated face database. All authors contributed to the article and approved the submitted version.

## Conflict of Interest

RR, DD, and CRY declare the existence of a financial competing interest in the form of a patent application based on this work. The remaining author declares that the research was conducted in the absence of any commercial or financial relationships that could be construed as a potential conflict of interest.

## Publisher’s Note

All claims expressed in this article are solely those of the authors and do not necessarily represent those of their affiliated organizations, or those of the publisher, the editors and the reviewers. Any product that may be evaluated in this article, or claim that may be made by its manufacturer, is not guaranteed or endorsed by the publisher.
