# Supplementary material for: Maximal Dependence Capturing as a Principle of Sensory Processing
Source: Front Comput Neurosci. 2022 Mar 25;16:857653. doi: 10.3389/fncom.2022.857653 (PMC8989953; doi:10.3389/fncom.2022.857653)
Supplement: Supplementary file 1 [file Data_Sheet_1.DOCX]

Supplementary Material

# Supplementary Figures


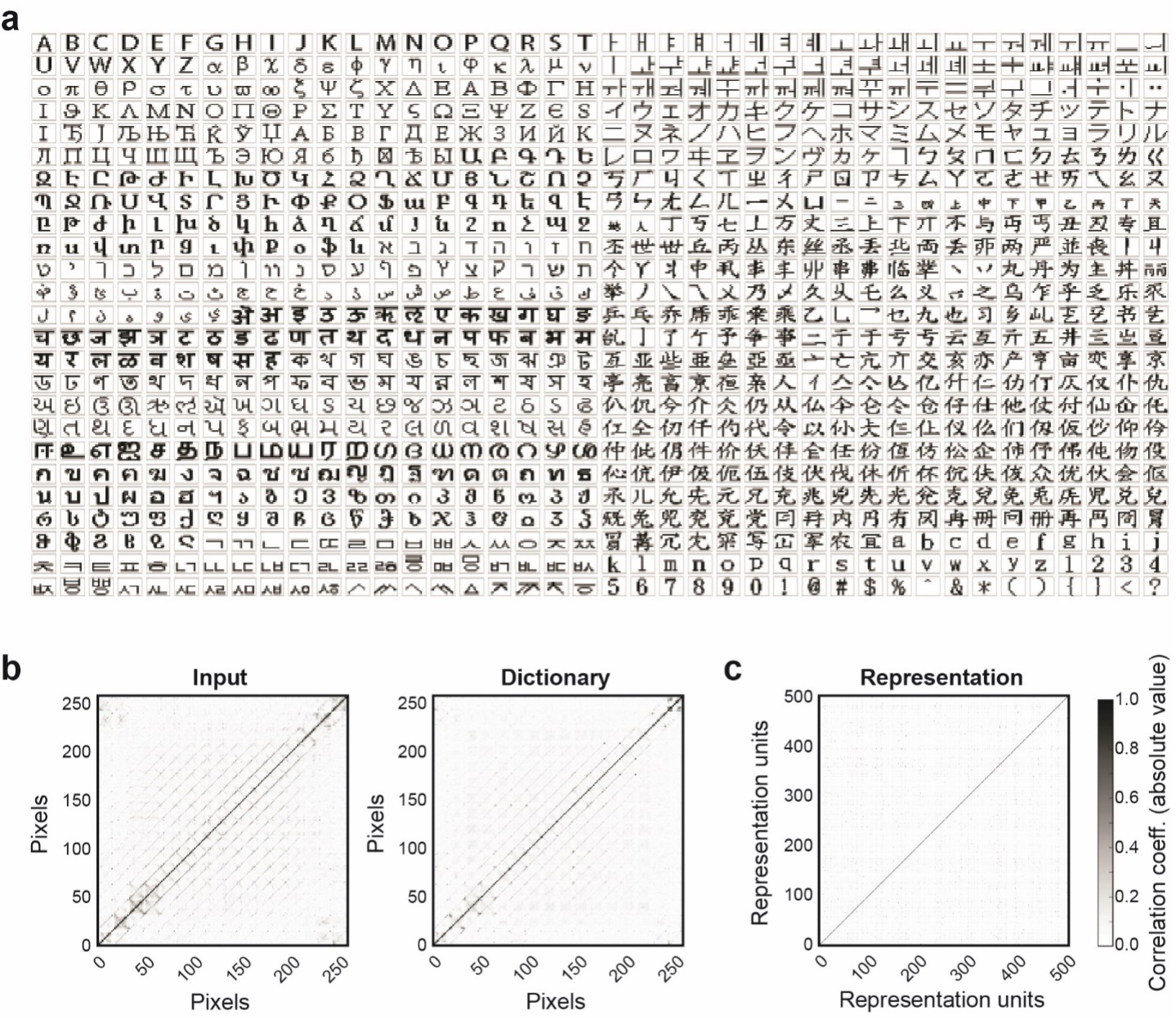


**Figure S1. Symbol representations using MDC framework.** (**A),** 1000 symbols used in the simulation. **(B),** Correlation between pixels as they occur in symbols and dictionary spaces. (**C),** Correlation between encoding units.


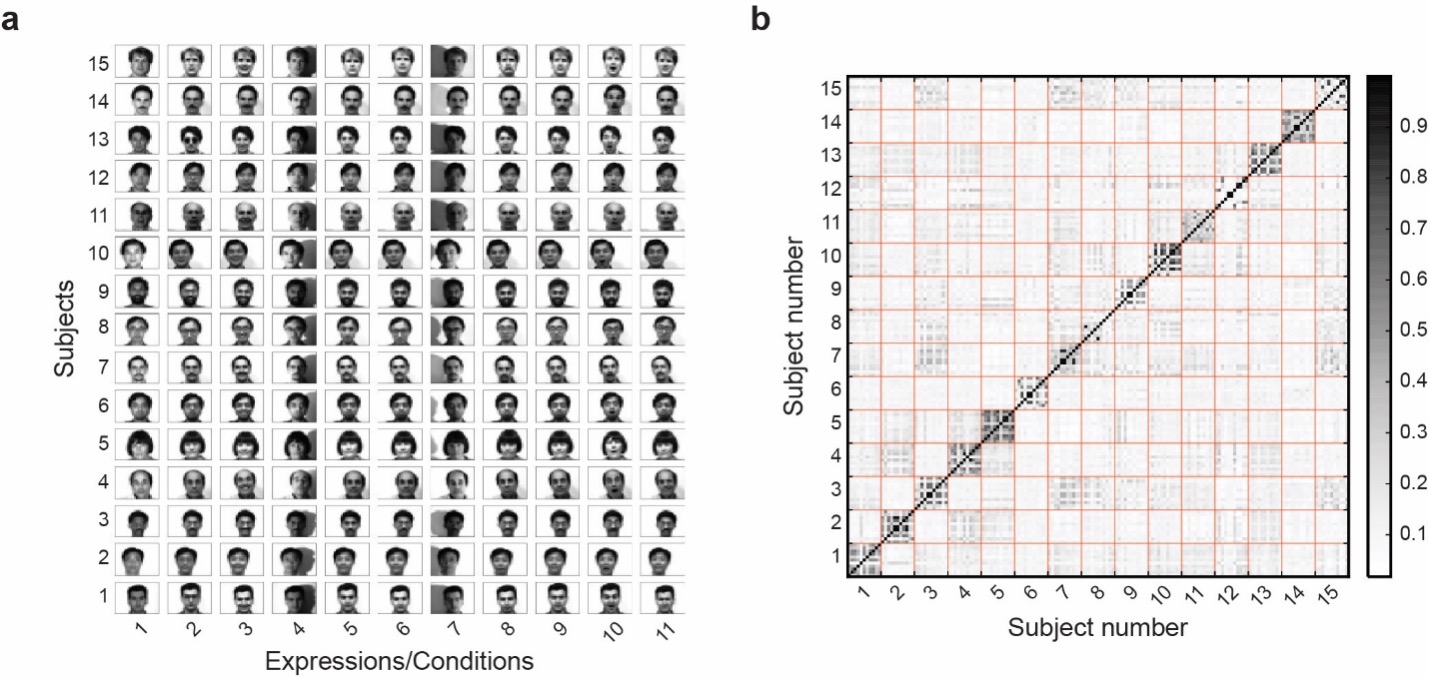


**Figure S2. Representation of faces from Yale database.** (**A),** images of 15 individuals with different lighting and facial expression**. (B),** Correlation between the output activities sorted according to individuals.


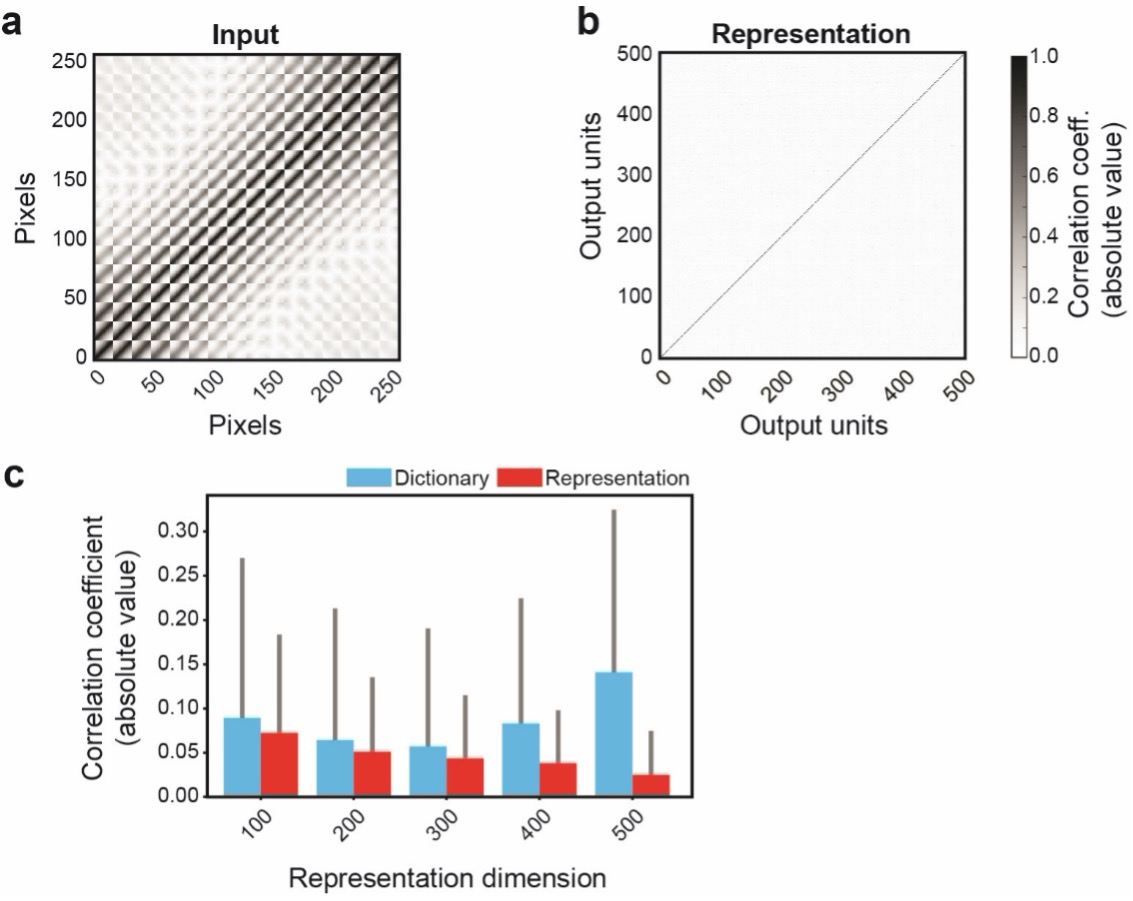


**Figure S3. Natural image analysis. (A),** Correlation among image pixels as they appear in images indicate high levels of redundancy. (**B),** Correlation among output units as they appear in the patches indicate decorrelation among representational units compared to image pixels. (**C),** Correlation among output units decrease as their numbers is increased. Bars indicate the mean of observed correlation coefficients (absolute values) and the whiskers indicate the observed standard deviation in the values.
